# Idiopathic Normal Pressure Hydrocephalus and Progressive Supranuclear Palsy: Two Single Entities or Neurodegenerative Overlap Syndrome? A Case Report

**DOI:** 10.3390/medicina59040720

**Published:** 2023-04-06

**Authors:** Igor Straka, Alice Martinkovicova, Michaela Jezberova, Tomas Zilka, Zuzana Kosutzka, Marian Saling, Peter Valkovic

**Affiliations:** 1 2nd Department of Neurology, Faculty of Medicine, Comenius University in Bratislava, University Hospital Bratislava, 813 72 Bratislava, Slovakia; 2 Department of Magnetic Resonance Imaging, Dr. Magnet Ltd., 833 05 Bratislava, Slovakia; 3 Department of Neurosurgery, Slovak Medical University, University Hospital–St. Michael’s Hospital, 811 08 Bratislava, Slovakia; 4 Institute of Normal and Pathological Physiology, Centre of Experimental Medicine, Slovak Academy of Sciences, 813 71 Bratislava, Slovakia

**Keywords:** idiopathic normal pressure hydrocephalus, neurodegenerative overlap syndrome, parkinsonism, progressive supranuclear palsy

## Abstract

The differential diagnosis of idiopathic normal pressure hydrocephalus (iNPH) and progressive supranuclear palsy (PSP) is difficult. The importance of proper diagnosis is particularly important for iNPH, which can be effectively treated with a ventriculoperitoneal (VP) shunt. In our case report, we present a unique case of a patient with overlapping symptoms and radiological findings of iNPH and PSP. Our patient underwent the VP shunt after a differential diagnostic evaluation which resulted in significant improvement in their clinical condition and quality of life, albeit for a short time.

## 1. Introduction

Idiopathic normal pressure hydrocephalus (iNPH) and progressive supranuclear palsy (PSP) are rare neurological disorders that primarily affect elderly people. However, some of the clinical features, especially gait/balance problems, and cognitive deficits are shared, which may complicate a differential diagnosis. The importance of a proper diagnosis is particularly important for iNPH, which can be effectively treated with a ventriculoperitoneal (VP) shunt. Here we present a case report of a patient with overlapping symptoms of iNPH and PSP.

## 2. Case Report

The patient was a male first diagnosed in February 2016 with Parkinson’s disease at the age of 65 by a neurologist (not a movement disorder specialist). The initial symptoms were bradykinesia and rigidity, predominantly on the right side, without cognitive deficits. After a year, he developed gait and balance disturbances, dementia, and urinary incontinence. The dopamine transporter SPECT (single-photon emission computed tomography) using ^123^I-Ioflupane (DATSCAN) (June 2017) showed a deficit of dopaminergic presynaptic transport in the area of the basal ganglia with asymmetrical involvement prominently on the left side.

The patient was Caucasian (white). According to his medical history, he was treated for arterial hypertension and hyperuricemia; he had vein thrombosis in the left eye in his youth. For the treatment, he had acetylsalicylic acid, bisoprolol, hydrochlorothiazide, and allopurinol. The patient had no history of neurological disorders himself nor in his family; there was no history of psychiatric disorders, and also no history of the use of neuroleptics and drug or alcohol addiction. The patient worked in the orchestra as a musician—a flutist.

**Figure 1:**
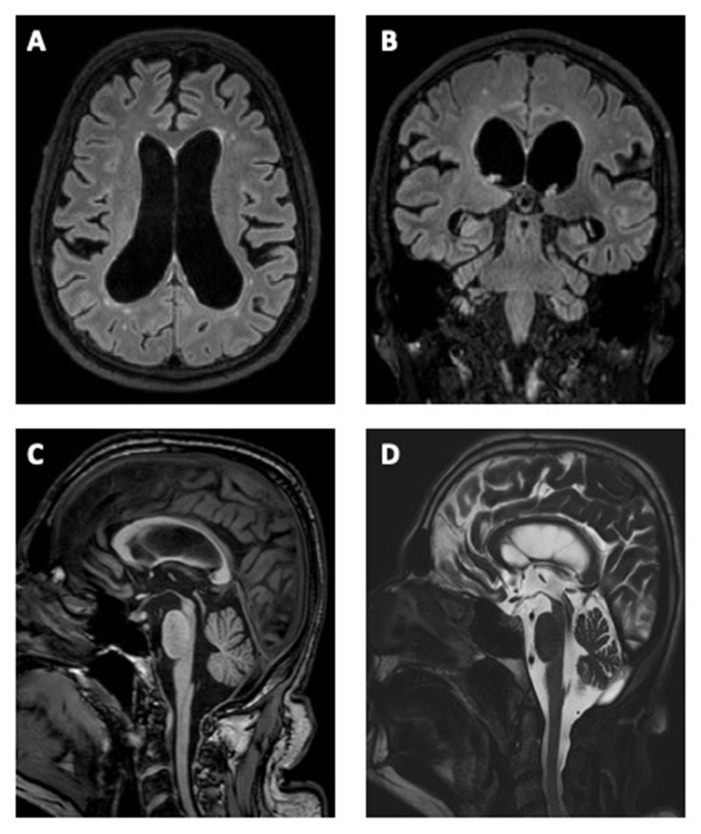
Brain MRI of the typical morphological changes associated with idiopathic normal pressure hydrocephalus and progressive supranuclear palsy. (**A**) Axial Flair, ventriculomegaly with an Evan’s index of more than 0.3, enlargement of lateral and third ventricles with a widening of the temporal horns, and mild T2 hyperintensities in the periventricular and deep white matter. (**B**) Coronal Flair, disproportionately enlarged subarachnoid spaces, tight high convexity (narrow sulci and subarachnoid spaces at the vertex and medial/parafalcine region) accompanied by enlargement of the inferior cerebrospinal fluid (CSF) spaces, particularly in the Sylvian fissures. The callosal angle measured at the level of posterior commissure is typically less than 80°. (**C**) Sagittal T1, midbrain atrophy (penguin or hummingbird sign), thinning and upward bowing of the corpus callosum. (**D**) Sagittal T2 Drive CSF—midbrain atrophy and strong flow void in the aqueduct of Sylvius.

The first examination at our Movement Disorder Center of the 2nd Department of Neurology, Comenius University and University Hospital Bratislava in May 2018 showed akinetic-rigid predominantly axial symmetric parkinsonism, impairment of saccades, and smooth pursuit eye movements mainly in the vertical direction with persevered oculocephalic reflex, hypokinetic dysarthria, dysexecutive syndrome, severe postural instability, and parkinsonian gait with a freezing of gait and falls (Supplementary Video S1). The patient was without any pyramidal signs, bulbar symptoms, sensorial cortical deficits, cerebellar signs, or autonomic dysfunction (except urinary incontinence which appeared after the first year after the onset of the parkinsonian symptoms). At this time, the patient was only able to walk unassisted when at home. The patient was treated with 0.18 mg pramipexole, three times a day (TID) and levodopa/carbidopa/entacapone 50/12.5/200 mg, four times a day (QID), without improvement of symptoms. Static posturography documented severe postural impairment and a positive Romberg test with backward falls. His posture was characterized by an increased amplitude of body sways. A neuropsychological examination revealed mild dementia, predominantly presenting as severe deficits of visuo-constructive, executive functions, with worsened performance in divided and selective attention; memory was globally deteriorated, mostly in the intelligibility and recognition memory (Montreal Cognitive Assessment = 19/30 points, Frontal Assessment Battery = 9/18 points). The patient had mild depression according to the Beck Depression Inventory—II (8/63 points). A brain MRI (magnetic resonance imaging) showed atrophy of the mesencephalon with hummingbird signs and ventriculomegaly. The Evans index was 0.33 and an MRI revealed an aqueduct flow void, disproportionately enlarged subarachnoid spaces, thinning and elevated corpus callosum with a callosal angle of 63°, and T2/Flair hyperintense lesions of white matter (Figure 1). Subsequently, we performed a cerebrospinal fluid (CSF) flowmetry MRI that showed the presence of hyperdynamic circulation through the aqueduct (stroke volume was 124 μL). Furthermore, the patient fulfilled the diagnostic criteria of clinical and MRI evidence for both of the two concurrent diagnoses—PSP (Level 2) and iNPH [1,2,3]. We performed a lumbar tap test with a withdrawal of 40 milliliters of CSF. In the three-meter Timed Up and Go Test, we observed a significant improvement in walking speed 24 h post lumbar puncture (17.5 vs. 13.2 s; Supplementary Video S2). In addition, the patient reported a significant improvement in walking and stability. The CSF examination revealed normal biochemical and cytological findings, with normal hTAU and pTAU181 levels (110.7 pg/mL; 35.1 pg/mL), and reduced Aß42 levels (394.4 pg/mL).

Based on these findings, we decided to perform VP shunt surgery (June 2018). Posturography two weeks after surgery revealed an improvement in posture, although when testing his stance on foam rubber he was still falling backward and the increased amplitude of body sways persisted. Overall, after the VP shunt the patient’s performance improved significantly, particularly in gait and balance, with unassisted mobility outdoors. Parkinsonism and oculomotor dysfunction persisted, cognitive functions did not deteriorate, and the incontinence disappeared. We adjusted the pharmacotherapy to levodopa/carbidopa 100/25 mg QID and amantadine 100 mg TID.

Six months after the VP shunt (December 2018), the patient’s balance and gait suddenly worsened due to thrombosis of the right transverse and sigmoid sinuses, with the patient showing gradual deterioration afterward. He was bedridden in March 2021 (34 months after the operation) and died in September 2021 as a result of severe urosepsis. Unfortunately, an autopsy was not performed on our patient due to the family’s disapproval.

## 3. Discussion

Idiopathic normal pressure hydrocephalus is an underdiagnosed and undertreated condition with a prevalence of 3.7% among individuals aged 65 years and older and is more common in the higher age groups [4]. The differential diagnosis of iNPH is difficult because the clinical symptoms can overlap with various other clinical entities and may be misdiagnosed as Alzheimer’s disease or other dementias (frontotemporal dementia or Lewy body dementia), Parkinson’s disease, atypical parkinsonian syndromes (mostly progressive supranuclear palsy), and vascular parkinsonism [5]. The diagnosis is made especially difficult by the presence of parkinsonian symptoms in the clinical picture, which is often, and may lead to an incorrect diagnosis [6,7,8,9,10]. This is particularly important because for patients with iNPH, treatment with a VP shunt leads to an improvement in the condition or a slowing down of the progression of the disease [11]. Therapy with a VP shunt has a success rate of up to 80% in patients and a relatively low rate of complications [12].

The primary question in our case report is whether the patient had a coincidence of iNPH and PSP, or a clinical phenotype of hydrocephalus with parkinsonism (PSP-like phenotype), or PSP, as some PSP patients develop iNPH-like MRI features and some iNPH patients develop PSP mimics [13]. The patient fulfilled the criteria for iNPH diagnosis with a complete Hakim’s triad [2,14,15], in parallel to possible PSP. Due to clinical diagnostic criteria, the patient met the criteria for PSP with Richardson’s syndrome in the core clinical features:

- ocular motor dysfunction (Level 2): slow velocity of vertical saccades,
- postural instability (Level 1): repeated unprovoked falls within three years,
- akinesia (Level 2): parkinsonism, akinetic-rigid, predominantly axial, and levodopa resistant,
- cognitive dysfunction (Level 2): frontal cognitive/behavioral presentation [1].

In an MRI, the callosal angle (specifically ≤71°) has the highest diagnostic accuracy to discriminate iNPH from its mimics, mostly from vascular dementia and atypical parkinsonism [16]. In our patient, the callosal angle was 63°. However, the possibility of a small callosal angle in PSP patients is also described in the literature, thus suggesting that the iNPH-like MRI features of PSP are not due to secondary ventriculomegaly induced by cortical atrophy. A longitudinal MRI study showed that disproportionately large ventricles for the degree of cortical atrophy can occur in PSP, which also indicates that a driving force other than cortical atrophy is associated with the enlarged ventricles in PSP [17]. Currently, PSP has no effective treatment and symptoms have limited dopaminergic responsivity. Similarly, as in PSP, some iNPH cases may also develop parkinsonian syndrome (including asymmetric presentation), which responds differently to shunt surgery. Possible mechanisms are a disconnection between the basal ganglia and supplementary motor area, imbalance at the dopaminergic-cholinergic level, a damaged nigrostriatal pathway, alteration of D2 receptors in the putamen, or pressure loading on the midbrain [18].

Another question is whether the patient should have been indicated for a VP shunt. After considering the significant response to the lumbar tap test, even with the eventual coincidence with PSP and the probability of improving the quality of life for a certain time, we decided to indicate a VP shunt after a thorough discussion with the patient and his family. The patient significantly benefited from the VP shunt for the next 6 months and we are convinced that the condition would have progressed more rapidly without surgical intervention. On the other hand, the thrombosis definitely worsened the prognosis and probably accelerated the process of neurodegeneration.

Therefore, the symptoms of different neurodegenerative disorders can overlap. In this case report, it was probable iNPH with parkinsonism (PSP-like phenotype); an autopsy would have helped to clarify the definite neuropathology process. However, in real clinical practice, it is most important to make a decision based on the clinical picture, especially when there are overlapping symptoms, because only an autopsy can provide a definitive diagnosis.

## 4. Conclusions

The differential diagnosis of iNPH and PSP is challenging and requires experienced movement disorder specialists. We reported on a patient with overlapping symptoms and the radiological findings of these two conditions after VP shunt surgery with a subsequent improvement in clinical condition. An autopsy was not performed on our patient. We would like to highlight the importance of proper clinical examination and differential diagnosis with regard to appropriate therapeutic procedures focusing on the improvement of the patient’s functional status and quality of life.

## Data Availability

Not applicable.

## Supplementary Materials

The following supporting information can be downloaded at: https://www.mdpi.com/article/10.3390/medicina59040720/s1, Video S1: Our patient, aged 67 years and presenting with akinetic-rigid predominantly axial symmetric parkinsonism, hypometric smooth pursuit eye movements predominantly with vertical movements, along with disturbance of both horizontal and vertical saccade movements with preserved oculocephalic reflex. Video S2: Lumbar TAP test with a withdrawal of 40 milliliters of cerebral spinal fluid evaluated in the three meters Timed Up and Go Test. Left: patient prior to lumbar puncture. Right: same patient 24 h after lumbar puncture.
